# Dual-Species Biofilms: Biomass, Viable Cell Ratio/Cross-Species Interactions, Conjugative Transfer

**DOI:** 10.3390/ijms241914497

**Published:** 2023-09-24

**Authors:** Marina V. Kuznetsova, Julia S. Pospelova, Irina L. Maslennikova, Marjanca Starčič Erjavec

**Affiliations:** 1 Institute of Ecology and Genetics of Microorganisms Ural Branch Russian Academy of Sciences, 614081 Perm, Russia; mar@iegm.ru (M.V.K.); i.maslennikova1974@gmail.com (I.L.M.); 2 LLC Centralized Clinical Diagnostic Laboratory, 614025 Perm, Russia; gizatullina.julia@yandex.ru; 3 Department of Microbiology, Biotechnical Faculty, University of Ljubljana, 1000 Ljubljana, Slovenia

**Keywords:** mixed biofilm, *Escherichia coli*, *Klebsiella pneumoniae*, *Enterococcus faecalis*, *Pseudomonas aeruginosa*, AI-2, conjugation

## Abstract

Biofilms as a form of adaptation are beneficial for bacterial survival and may be hot spots for horizontal gene transfer, including conjugation. The aim of this research was to characterize the biofilm biomass, viable cell ratios and conjugative transfer of the pOX38 plasmid, an F-plasmid derivative, from the *Escherichia coli* N4i pOX38 strain (donor) into a uropathogenic *E. coli* DL82 strain (recipient) within dual-species biofilms with one of the following opportunistic pathogenic bacteria: *Klebsiella pneumoniae*, *Enterococcus faecalis* or *Pseudomonas aeruginosa*. Dual-species biofilms of *E. coli* with *K. pneumoniae* or *P. aeruginosa* but not *E. faecalis* were more massive and possessed more exopolysaccharide matrix compared to single-species biofilms of donor and recipient cells. Correlation between biofilm biomass and exopolysaccharide matrix was rs = 0.888 in dual-species biofilms. In dual-species biofilm with *E. faecalis* the proportion of *E. coli* was the highest, while in the biofilm with *P. aeruginosa* and *K. pneumoniae*, the *E. coli* was less abundant. The conjugative frequencies of plasmid transfer in dual-species biofilms of *E. coli* with *E. faecalis* and *P. aeruginosa* were reduced. A decrease in conjugative frequency was also observed when cell-free supernatants (CFSs) of *E. faecalis* and *P. aeruginosa* were added to the *E. coli* conjugation mixture. Further, the activity of the autoinducer AI-2 in the CFSs of the *E. coli* conjugation mixture was reduced when bacteria or CFSs of *E. faecalis* and *P. aeruginosa* were added to the *E. coli* conjugation mixture. Hence, the intercellular and interspecies interactions in dual-species biofilms depend on the partners involved.

## 1. Introduction

It is well known that biofilms are a natural form of existence for most bacteria in natural ecosystems and human biotopes [1,2]. Biofilms on biotic and abiotic surfaces are a major problem in medical, environmental, industrial and agricultural settings [3,4,5]. As bacteria in biofilms have an increased resistance to antibiotics, biofilms, including multi-species biofilms, pose a health risk in medical practice. In addition, biofilms can be reservoirs of bacteria that can cause constant reinfection and chronic inflammation, which can also lead to tissue damage, clogging of devices and general resistance to treatment [6,7]. In biofilms associated with urinary tract infections (UTIs), *Escherichia coli* strains are most prevalent, but other commensals and opportunistic pathogens such as *Klebsiella pneumoniae*, *Enterococcus faecalis* and *Pseudomonas aeruginosa* can also be found [8,9]. Bacterial communication within the biofilm allows them to orchestrate the expression of virulence genes, which further cements the infestation and increases the invasiveness of the infection [10]. These facts emphasize the need to study biofilms of uropathogenic bacteria by describing the mechanisms of interspecies and intraspecies interactions that underlie the formation and functioning of polymicrobial biofilms.

There are a number of studies on the mutual influence of opportunistic pathogenic bacteria on the formation of polymicrobial biofilms. Oliveira et al. (2018) showed that the number of *E. coli* cells in a mixed biofilm formed during co-cultivation with *P. aeruginosa* could be higher or lower than in a single-species one, depending on the surface on which the bacteria formed the biofilm (polystyrene 96-well microtiter plate vs. porcine skin explants, respectively) [11]. It was found that the viability of *E. coli* cells in biofilm is suppressed by *K. pneumoniae* [12]. Further, it has also been shown that the presence of *E. coli* can interfere with the attachment of *E. faecalis* to the surface, slowing the development of a mixed biofilm [13]. Similar data have been obtained with clinical isolates of *E. coli* which secreted polysaccharides that altered the properties of abiotic surfaces, thereby inhibiting biofilm formation of Gram-positive bacteria, including *E. faecalis* [14].

Bacterial communication is important both in competition and in synergistic relationships that lead to enhanced metabolic cooperation. The most studied form of cell interaction in bacterial communities is known as quorum sensing (QS). QS response is mediated by different extracellular autoinducer molecules, among which is AI-2 (autoinducer 2; LuxS), a furanosyl borate diester which is an interspecies autoinducer. Its gene *luxS* is present in many bacterial species (*E. coli*, *K. pneumoniae*, *E. faecalis*). It is known that AI-2 affects virulence and biofilm formation of *E. coli*, *K. pneumoniae*, *E. faecalis* and *P. aeruginosa* [15,16,17,18,19,20], but the role of AI-2 in mixed cultures is not well understood [21,22].

Interspecies interactions within the biofilm affect the growth and survival of all members, their cellular metabolic processes, and the intra- and interspecific exchange of genetic information. Interspecies interactions therefore affect the development, structure, and functions of multispecies biofilms [23,24,25]. Quorum-sensing autoinducer molecules produced by members of a multispecies biofilm promote horizontal gene transfer [26], which is important for the emergence of multiple drug-resistant bacteria associated with the widespread use of antibiotics in medicine and veterinary medicine [27]. F- and F-like plasmids are of great importance for the maintenance and dissemination of the ecophysiological traits of bacteria: growth, reproduction rate, biofilm formation and resistance to antibiotics and bacteriocins [28,29,30]. Transfer of the conjugative pOX38 plasmid into UPEC strains has been shown to depend on biofilm biomass [31]. It is assumed that the presence of opportunistic pathogenic bacteria or their cell-free supernatants (CFSs) may disrupt the optimal ratio of donor and recipient cells and may affect the efficiency of horizontal gene transfer.

The aim of this work was to characterize the biofilms’ biomass, viable cell ratios/cross-species interactions and conjugative transfer abilities in dual-species biofilms of *E. coli* with following opportunistic pathogenic bacteria: *K. pneumoniae, P. aeruginosa* and *E. faecalis.*

## 2. Results

### 2.1. Biofilm Biomass

**Figure 1:**
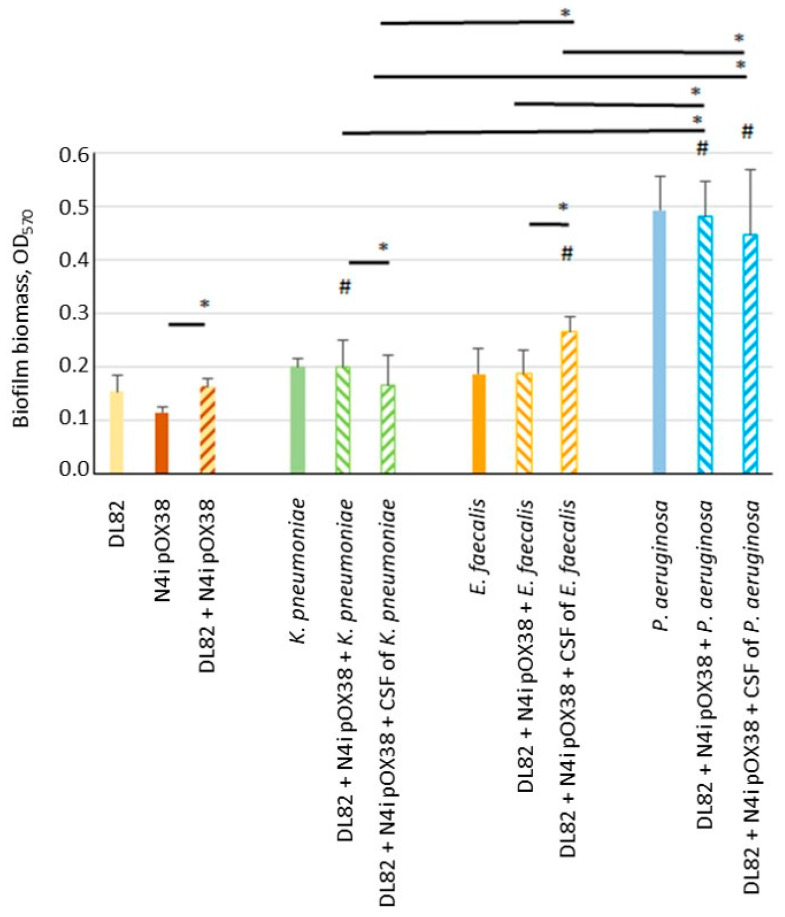
Biofilm biomass of recipient *E. coli* DL82, donor *E. coli* N4i pOX38, *K. pneumoniae*, *E. faecalis* and *P. aeruginosa* in single-species biofilms, dual-species biofilms, and under influence of cell-free supernatants (CFSs) of opportunistic pathogenic bacterial species. ^#^—statistically significant difference compared to the biofilm biomass of the DL82+ N4i pOX38 single-species biofilm (*t*-test; *p* < 0.05); *—statistically significant difference between denoted values (*t*-test; *p* < 0.05).

The determined biofilm biomass is shown in Figure 1. The biomass of single-species biofilms of the two *E. coli* strains, recipient DL82 (R) and donor N4i pOX38 (D), *K. pneumoniae* and *E. faecalis* were similar to each other and OD_570_ ranged from 0.1 to 0.2. The biofilm biomass formed by *P. aeruginosa* was significantly higher and almost reached, on average, an OD_570_ of 0.5 (*p* = 0.01). The dual-species biofilms of *E. coli* with *K. pneumoniae* and *E. coli* with *P. aeruginosa* were significantly more massive than the single-species *E. coli* biofilm (R + D) (*p* = 0.022 and *p* = 0.0001, respectively). In cases where, instead of the cells of opportunistic species, CFSs of opportunistic pathogenic bacteria were added, the *E. coli* biomass was significantly more massive with the CFSs of *E. faecalis* and *P. aeruginosa* than it was with the single-species *E. coli* biofilm (R + D) (*p* = 0.0001 and *p* = 0.0001, respectively). Cell-free supernatant (CFS) of *K. pneumoniae* as well as CFS of *P. aeruginosa* decreased the biofilm’s biomass, while the CFS of *E. faecalis* increased the biofilm’s biomass when compared with dual-species biofilms of *E. coli* with opportunistic pathogenic bacteria (*p* = 0.026 and *p* = 0.0001, respectively). Among studied opportunistic potentially pathogenic bacterial species, *P. aeruginosa* cells and the *P. aeruginosa* CFS, when added to *E. coli* (R + D), contributed to a much higher increase in the biofilm’s biomass compared to *K. pneumoniae* and *E. faecalis* cells and CFSs (*p* = 0.0001).

### 2.2. Biofilm Exopolysaccharide Matrix

**Figure 2:**
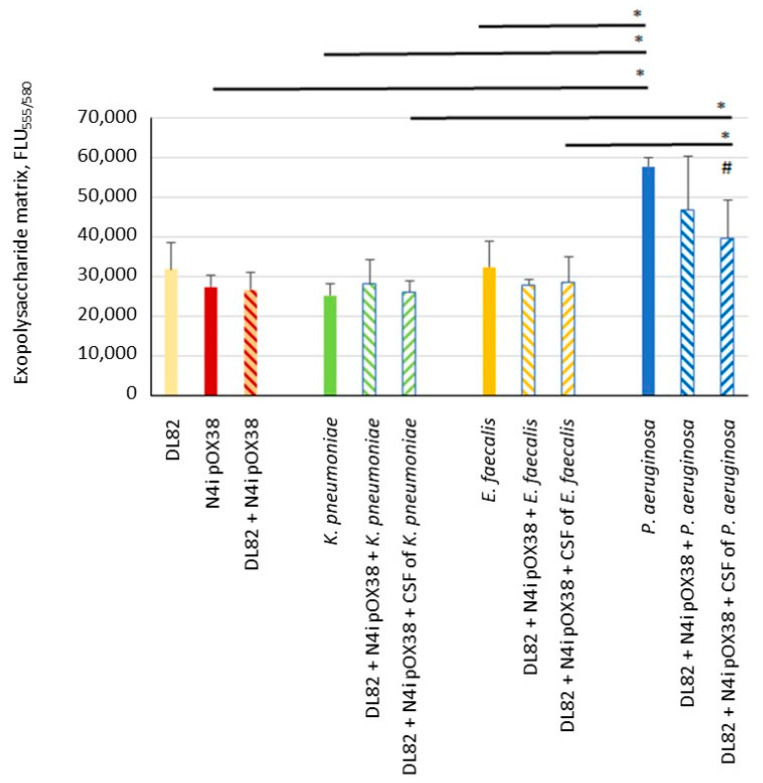
The exopolysaccharide of recipient *E. coli* DL82, donor *E. coli* N4i pOX38, *K. pneumoniae*, *E. faecalis* and *P. aeruginosa* in single-species biofilms, dual-species biofilms, and under influence of CFSs of opportunistic pathogenic bacterial species. ^#^—statistically significant difference compared to the control (DL82+ N4i pOX38) (*t*-test; *p* < 0.05); *—statistically significant difference between denoted values (*t*-test; *p* < 0.05).

The determined massiveness of the exopolysaccharide matrix of biofilms is shown in Figure 2. The exopolysaccharide matrix massiveness of single-species biofilms of the two *E. coli* strains, recipient DL82 and donor *E. coli* N4i pOX38, and two opportunistic pathogenic bacteria, *K. pneumoniae* and *E. faecalis*, were similar to each other, and FLU_555/580_ ranged from 25,000 to 32,000. The exopolysaccharide matrix massiveness of the biofilm formed by *P. aeruginosa* was significantly higher than that of the matrix of donor *E. coli* N4i pOX38, *K. pneumoniae* and *E. faecalis*, and almost reached an FLU_555/580_ of 60,000 (*p* = 0.001). The donor and the recipient exopolysaccharide amounts did not differ in single- and mixed-species biofilms, except for the exopolysaccharide amount of the biofilm formed when *P. aeruginosa* or its CFS was added. The correlation between biofilm biomass and exopolysaccharide matrix was rs = 0.888, indirectly indicating that in highly exopolysaccharide-producing bacteria, the matrix plays a more important role in biofilm biomass than do the cells.

### 2.3. The Viability of Recipient E. coli DL82, Donor E. coli N4i pOX38 and Opportunistic Pathogenic Bacteria within Dual-Species Biofilms 

**Figure 3:**
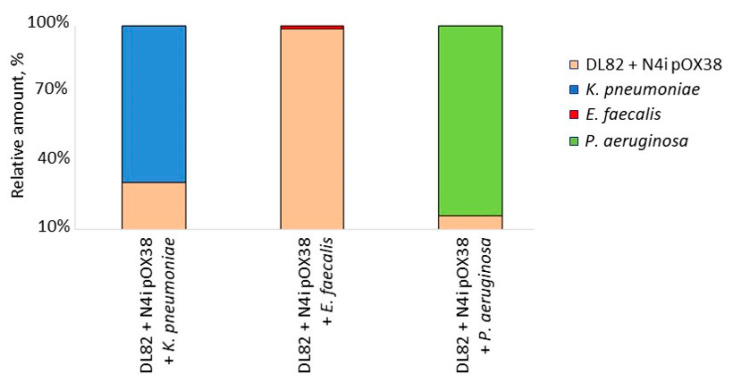
Ratio of *E. coli* (DL82+ N4i pOX38) and opportunistic pathogenic bacterial species (*K. pneumoniae*, *E. faecalis*, *P. aeruginosa*) in dual-species biofilms.

The ratio of viable *E. coli*-opportunistic pathogenic bacterial cells in biofilms of dual-species cultures after 24 h differed in biofilms with different opportunistic pathogenic bacteria (Figure 3), even though all cultures initially started with the same ratio of *E. coli*-opportunistic pathogenic bacteria. After 24h incubation, the proportion of *E. coli* was the highest (98.76%) in the dual-species biofilm with *E. faecalis*, while in the biofilm with *P. aeruginosa*, there was a proportion of only 16.38% *E. coli* cells. *E. coli* was also less abundant in the dual-species biofilm formed with *K. pneumoniae* (30.79%).

**Figure 4:**
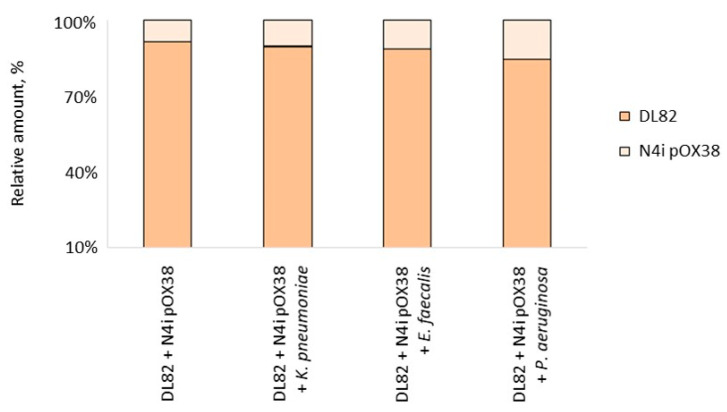
Ratio of recipient *E. coli* DL82 and donor *E. coli* N4i pOX38 in studied single- and dual-species biofilms with opportunistic pathogenic bacterial species (*K. pneumoniae*, *E. faecalis*, *P. aeruginosa*).

It should be noted that despite the different proportion of *E. coli* in the studied dual-species biofilms, the ratios of recipient and donor cells in the studied biofilms did not change significantly (Figure 4). 

### 2.4. The Frequency of Conjugation within Dual-Species Biofilm

**Figure 5:**
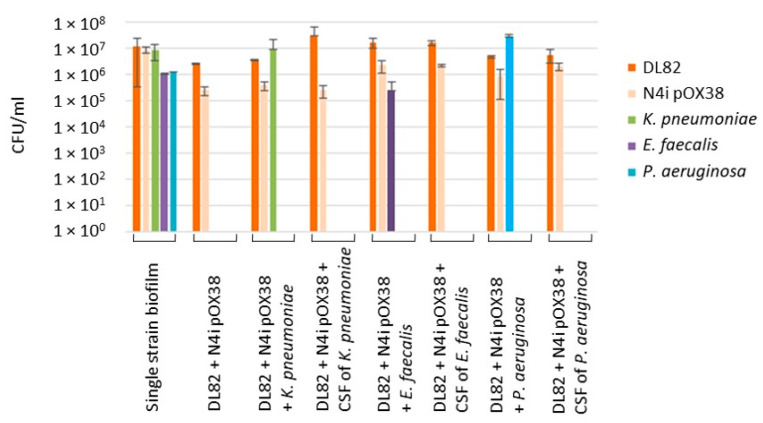
CFU data of recipient *E. coli* DL82, donor *E. coli* N4i pOX38 and opportunistic pathogenic bacteria *K. pneumoniae*, *E. faecalis* and *P. aeruginosa* in biofilms in conjugation assays.

The CFU data used to calculate the conjugation frequency are shown in Figure 5. There were no statistically significant differences in the CFUs of recipient *E. coli* DL82 and donor *E. coli* N4i pOX38 in single-species biofilms, dual-species biofilms with opportunistic pathogenic bacteria, or in single-species biofilms with added CFSs of opportunistic pathogenic bacteria (Figure 5). There was also no statistically significant difference in the CFUs of opportunistic pathogenic bacteria in single-species biofilms compared to dual-species biofilms (Figure 5).

The frequencies of conjugative transfer of the F-plasmid derivative from the donor *E. coli* N4i pOX38 (D) into the recipient *E. coli* DL82 (R) in biofilms are shown in Table 1. As can be seen from Table 1, the conjugative frequencies of plasmid transfer in dual-species biofilms (*E. coli* (R + D) + *E. faecalis*/*P. aeruginosa* cells) were reduced (U-test: *p* = 0.0495) in contrast to the *K. pneumoniae* dual-species biofilm. Addition of CFSs of opportunistic pathogenic bacteria (*K. pneumoniae* or *E. faecalis*) to the conjugation mixture resulted in lower conjugative frequencies (decreased by about 1.5 orders); conjugation was completely absent in the presence of *P. aeruginosa* CFS.

Statistical analysis revealed several negative correlations: between conjugation frequency and total biofilm biomass (rs = −0.440), between conjugation frequency and exopolysaccharide matrix of biofilms (rs = −0.270), between conjugation frequency and CFU of *E. coli* in biofilm (rs = −0.390) and between conjugation frequency and the total CFU number (R + D + opportunistic pathogenic bacteria) (rs = −0.713).

### 2.5. AI-2 Activity in CFSs of Conjugation Mixtures

The results of an AI-2 production assay are given in Table 2. It was shown that donor *E. coli* N4i pOX38 and recipient *E. coli* DL82 produced a low amount of AI-2 when grown in single-species culture, while in the conjugation mixture (R + D), the production level of AI-2 increased, but did not exceed, the activity of the positive control BB152 strain. The AI-2 activity in the *K. pneumoniae* CFS was higher than in CFS of *E. coli* strains: DL82 and N4i pOX38; however, the activity of AI-2 did not change in the CFSs of the conjugation mixture (R + D) in the presence of *K. pneumoniae* cells or CFS of *K. pneumoniae*. CFSs of single-species cultures of *P. aeruginosa* and *E. faecalis* did not show any autoinducer activity. However, the presence of opportunistic pathogenic bacteria or CFS of these species in the conjugation mixture reduced the activity of the autoinducer (Table 2) when compared to the control (R + D). 

## 3. Discussion

The ecological advantages of microorganisms in biofilms are associated with protection from adverse environmental conditions, biocides and immune effectors, and enhanced metabolic cooperation. Communication between cells of a single-species biofilm mediated by bacterial metabolites (QS regulation systems) can change the expression of genes that provide control over motility, adhesion, biofilm formation and virulence of bacteria, as well as the exchange of genetic information [15,16,17,18,19,20,26,32]. The universal signal molecule AI-2 is well known to regulate inner- and interspecies cell-density-dependent phenotypes, but the role of AI-2 in the establishment of multispecies communities is not well understood [22]. The LuxS/AI-2 system has been implicated in the regulation of numerous bacterial features, including biofilm formation and motility as well as horizontal gene transfer [21]. The low AI-2 level in the media during growth of the recipient and donor *E. coli* strains in a single culture is probably related to its consumption by the bacteria themselves during the 24 h cultivation. On the other hand, it is known that *E. coli* strains can take up autoinducers during coexistence [33], which was confirmed in our study, as an increase in the AI-2 level was observed in the mixed culture (R + D), probably due to the blocking of autoinducer transport systems of the competitive strain.

There are two main components in the biofilm structure: bacterial cells and an extracellular matrix; these unite microbial cells into a single system, the architecture of which ensures the efficiency of intercellular contacts during conjugative transfer [34]. Due to close cell-to-cell contact and the presence of DNA and signaling molecules, biofilms could be hot spots for horizontal gene transfer [35]. F-plasmids of *E. coli* are the most common conjugative plasmids associated with recombinant antibiotic resistance (“R-factors”) [29] and known to be efficiently transferred in biofilms [36,37].

It is well known that uropathogenic bacteria have high adhesion and biofilm-forming ability, which leads to chronic recurrent infections of the urinary system. In addition, the frequent use in urologic practice of biologics and artificial materials, on the surface of which bacterial biofilms form, increases the risk of persistence of the pathogen in the body [9]. For example, UPEC strains have a clear competitive advantage during biofilm growth on catheter surfaces [38]. *E. coli*, *K. pneumoniae*, *P. aeruginosa* (Gram-negative bacteria) and *E. faecalis* (Gram-positive bacteria) are known inhabitants of the human gastrointestinal tract, which have been found to co-occur in catheter-associated urinary tract infections too [8]. In the present work, interspecies interactions in dual-species biofilms formed by *E. coli* and either *K. pneumoniae*, *E. faecalis*, or *P. aeruginosa* were studied. 

Our study showed that the CFU of *E. coli* (R + D) did not change in dual-species biofilms of *E. coli* with *K. pneumoniae* when compared to single-species *E. coli* biofilm (R + D) (Figure 5). This result is in contrast to previous data showing that viability of *E. coli* in biofilm is suppressed by *K. pneumoniae* [12]. Interestingly, despite a noticeably lower ratio of *E. coli* in the biofilm during co-growth with *K. pneumoniae* (Figure 3), the frequency of intraspecific conjugation decreased only slightly (Table 1), which may be due to the preserved donor-to-recipient ratio (Figure 3 and Figure 4) and the spatial structure of biofilms with a less-pronounced exopolysaccharide matrix component in biofilms of these bacterial species (Figure 2). In our study, no effect of *Klebsiella* CFS on *E. coli* conjugation in biofilm or on AI-2 activity in CFSs of conjugation mixture (Table 2) was detected. Recently, also, Juarez and Galván (2018) [12] in their study showed that *Klebsiella* CFS had no pronounced antimicrobial or “antibiofilm” effect on *E. coli*.

It is known that when *E. coli* is co-cultured with *E. faecalis*, the latter significantly enhances biofilm formation and viability of *E. coli* in vitro and in vivo [39]. This was also observed in our study (Figure 3). The observed effect may be due to *E. faecalis* exporting L-ornithine, which facilitates biosynthesis of *E. coli* enterobactin siderophores, enabling *E. coli* growth and biofilm formation under iron-limiting conditions [39]. On the other hand, *E. faecalis* is known to be an active producer of AI-2, which stimulates the coaggregation of *E. coli* cells, leading to the formation of a large number of aggregates of both cell types [22]. In our study, the level of AI-2 was low in the CFS of *E. faecalis* and in the conjugation mixture of *E. coli* with this opportunistic pathogenic bacterium, whereas the AI-2 level was higher in the conjugation mixtures with the *E. faecalis* CSF (Table 2). This may be due to subsequent consumption of AI-2 after 24 h. The low level of AI-2 might affect the conjugative contact efficiency of *E. coli* strains, leading to the observed decrease in conjugative frequency (Table 1), although the number of *E. coli* cells, as well as the biomass of the mixed biofilm, were comparable to those of the control (Figure 1 and Figure 5). 

Previous studies showed contrasting results of co-culture of *E. coli* with *P. aeruginosa*. Some researchers reported that the biomass in the mixed biofilm biomass did not increase, compared to *E. coli* single culture [40,41], while others reported significantly more-massive biofilms and higher cell numbers of both taxa in dual-species biofilms compared to single-species biofilms [11,42]. Interestingly, indole, which is formed during protein hydrolysis and is the most important extracellular regulator of the *Escherichia* population, stimulates the formation of surface biofilms of *Pseudomonas putida* [43]. Many studies have shown that *P. aeruginosa* inhibits *E. coli* viability during co-growth [11,44,45], which is consistent with our research findings (Figure 3 and Figure 5), in which, similar to the dual-species biofilm with *K. pneumoniae*, most cells in the dual-species biofilm with *P. aeruginosa* were *P. aeruginosa* cells, but the efficiency of conjugative transfer was significantly lower in the mixed conjugation mixture of *E. coli* with *P. aeruginosa* (Table 1). The reason may relate to the influence of CFSs. On one hand, previous data demonstrated that planktonic and biofilm exoproducts of *P. aeruginosa* did not affect the plankton growth and biofilm formation of *E. coli* [40], but on the other hand, in our study the *E. coli* biofilm and biofilm exopolysaccharide matrix formed in the presence of *P. aeruginosa* CFS were more massive (Figure 1 and Figure 2). Due to limited diffusion to the outside, the biofilm matrix acts as a molecular reservoir, so molecules released by antagonistic bacteria in the biofilm become more concentrated in local areas of the biofilm, resulting in increased deleterious effects on *E. coli*. In the same way, diffusion of AI-2 molecules in mixed biofilms of *E. coli* with *P. aeruginosa* may be prevented, resulting in lower AI-2 activity compared to single-species biofilms (Table 2). A more massive matrix can also prevent cell contact, including mating pair formation, such that lower conjugative frequencies of plasmid transfer are obtained (Table 1). It should be noted that in the present study, complete inhibition of the plasmid transfer process was observed after *E. coli* exposure to *P. aeruginosa* CFS.

Interestingly, in experiments with artificial introduction of AI-2 or its precursor analogues (DPD) into the culture medium, an increase in biofilm mass [46] and a decrease in expression of genes associated with conjugation were observed [47]. On the contrary, Cho et al. (2003) showed a positive correlation between conjugation efficiency and increasing AI-2 levels [48], supporting the hypothesis that AI-2 plays a positive regulatory role in bacterial conjugations. Our studies revealed a trend towards a positive correlation between the AI-2 level in the culture medium and the conjugative frequency when adding CFSs of opportunistic pathogens (r = 0.45) or their bacterial cells (0.89). Thus, the ability of AI-2 to regulate horizontal gene transfer appears to be justified under conditions of interspecific competition in microbial communities.

## 4. Materials and Methods

### 4.1. Strains and Media

The uropathogenic *E. coli* DL82 (Amp^r^) deposited in the collection of the University of Ljubljana [49] was used as the recipient in conjugation assays. *E. coli* N4i pOX38 (Gen^r^ Cm^r^), constructed on the basis of *E. coli* Nissle 1917 by introducing the conjugative pOX38 plasmid (a derivative of the *E. coli* K12 F plasmid) [50], was used as the donor in conjugation assays. Wild type *E. coli* K12, *Klebsiella pneumoniae* ATCC^®^700603 and *Pseudomonas aeruginosa* ATCC^®^27853, obtained from “Scientific Centre for Expert Evaluation of Medicinal Products” of the Ministry of Health of the Russian Federation and a clinical strain of *Enterococcus faecalis* isolated from a fecal sample of a patient with intestinal infection (Perm, Russian Federation) were used as opportunistic pathogenic bacteria to form dual-species biofilms with *E. coli.* All strains were grown in Luria-Bertani (LB) medium (Amresco, Solon, OH, USA). The AI-2 production assay strains *Vibrio harveyi* BB170 (*luxN*::Tn*5*, sensor AI-2) and BB152 (*luxL*::Tn*5*, AI-2 producer) [51] were grown in autoinducer bioassay medium (AB) consisting of 17.5 g/L NaCl, 12.3 g/L MgSO_4_, 2.0 g/L casamino acids (vitamin-free), 1 M KH_2_PO_4_ (pH 7.0), 0.1 M L-arginine and 10 mL/L glycerol [51]. 

### 4.2. Cell-Free Supernatant (CFS)

Bacterial cultures were grown for 24 h at 37 °C in LB medium. After the 24-h incubation, 100 µL of bacterial cultures were centrifuged at 8000 rpm (Eppendorf, Hamburg, Germany) for 10 min and then filtered through a Millex^®^-GS membrane filter (Merck Milli-pore Ltd., Carrigtwohill, Ireland) with a pore size of 0.22 µm to obtain sterile CFSs. The sterility of the CFSs was tested by direct inoculation on LB-agar.

### 4.3. Conjugation Assay

The overnight cultures of *E. coli* DL82, *E. coli* N4i pOX38, *K. pneumoniae*, *E. faecalis* and *P. aeruginosa* were diluted 1:100 in fresh LB media and grown for 3h at 37 °C with shaking (180 rpm). Then, the following conjugation mixtures were prepared: (1) 80 µL of the recipient (*E. coli* DL82 (Amp^r^); 10^6^ cells/mL), 40 µL of donor (*E. coli* N4i pOX38 (Gen^r^ Cm^r^) 2 × 10^6^ cells/mL) and 80 µL of fresh LB (control); (2) 80 µL of the recipient (*E. coli* DL82 (Amp^r^) 10^6^ cells/mL), 40 µL of donor (*E. coli* N4i pOX38 (Gen^r^ Cm^r^); 2 × 10^6^ cell/mL) and 80 µL opportunistic pathogenic bacteria (10^6^ cells/mL) (experimental model 1); and (3) 80 µL of the recipient (*E. coli* DL82 (Amp^r^) 10^6^ cells/mL), 40 µL of donor (*E. coli* N4i pOX38 (Gen^r^ Cm^r^) 2 × 10^6^ cell/mL) and 80 µL of CSFs of opportunistic pathogenic bacteria (experimental model 2). A quantity of 100 µL of the conjugation mixture was added to a 96-well flat bottom polystyrene plate (Medpolimer, Saransk, Russia) and incubated for 24 h at 37 °C without shaking. After the 24 h incubation the 96-well flat bottom plate was washed three times with NaCl (0.9%) and then sonicated (5 times for 1 min at 37 kHz) in an Elma Ultrasonic 30S ultrasonic bath (Elma, Berlin, Germany) and bacterial suspensions were plated on: LB supplemented with chloramphenicol (50 µg/mL) and ampicillin (50 µg/mL) (for transconjugant CFU count); LB supplemented with ampicillin (50 µg/mL) (for recipient CFU count); LB supplemented with gentamicin (40 µg/mL) (for donor CFU count); MacConkey-GRM agar (Federal Scientific Research Center of PMB, Obolensk, Russia) (for *K. pneumoniae* CFU count); cetrimide agar (Federal Scientific Research Center of PMB, Obolensk, Russia) (for *P. aeruginosa* CFU count); and enterococcus selective agar (Federal Scientific Research Center of PMB, Obolensk, Russia) (for *E. faecalis* CFU count). All plates were grown 24 h at 37 °C. After the incubation the CFUs were counted and the frequency of conjugation was calculated as the ratio of the number of CFUs of transconjugants and the number of CFUs of recipients [52]. Experiments were made three times in three technological repeats.

### 4.4. Biofilm Biomass Analysis

Biofilm biomass formed by single-species culture, dual-species culture (*E. coli* with opportunistic pathogenic bacteria) or single-species culture with added CFSs (*E. coli* and CFSs of opportunistic pathogenic bacteria) was determined. The overnight cultures of *E. coli* DL82, *E. coli* N4i pOX38, *K. pneumoniae*, *E. faecalis* and *P. aeruginosa* were diluted 1:100 in fresh LB media and grown for 3h at 37 °C with shaking (180 rpm). Then, the following samples were prepared: (1) 80 µL of the recipient (*E. coli* DL82 (Amp^r^); 10^6^ cells/mL), 40 µL of donor (*E. coli* N4i pOX38 (Gen^r^ Cm^r^) 2 × 10^6^ cells/mL) and 80 µL of fresh LB (control); (2) 80 µL of the recipient (*E. coli* DL82 (Amp^r^); 10^6^ cells/mL), 40 µL of donor (*E. coli* N4i pOX38 (Gen^r^ Cm^r^) 2 × 10^6^ cell/mL) and 80 µL opportunistic pathogenic bacteria (10^6^ cells/mL) (experimental model 1); and (3) 80 µL of the recipient (*E. coli* DL82 (Amp^r^) 10^6^ cells/mL), 40 µL of donor (*E. coli* N4i pOX38 (Gen^r^ Cm^r^) 2 × 10^6^ cell/mL) and 80 µL of CSF of opportunistic pathogenic bacteria (experimental model 2). A quantity of 100 µL of the prepared samples was added to wells of a 96-well flat bottom polystyrene plate (Medpolimer, Saint Petersburg, Russia) and incubated for 24 h at 37 °C without shaking. The biofilm biomass was determined according to Merritt J. H. et al. (2005) [53]. The formed biofilms were washed three times with NaCl (0.9%), stained with 0.1% gentian violet for 30 min and washed twice with distilled water, followed by elution of the dye with 200 μL of 96% ethanol to determine biofilm biomass by optical density (OD_570_) using an Infinite M1000 (TECAN, Grödig, Austria). Experiments were made three times in three technological repeats.

### 4.5. Biofilm Exopolysaccharide Matrix Analysis

Single-species culture, dual-species culture (*E. coli* with opportunistic pathogenic bacteria) or single-species culture with added CFSs (*E. coli* and CFSs of opportunistic pathogenic bacteria) were grown in the wells of a black 96-well plate (Nunc, Roskilde, Denmark) for 24 h at 37 °C without shaking, as stated in the Biofilm Biomass Analysis section. After incubation and washing three times with NaCl (0.9%), the biofilms were stained with 100 μL of conA-tetramethylrhodamine solution (500 μg/mL) for 40 min in the dark; subsequently, the dye was removed and the biofilm was washed once with 100 µL 0.9% NaCl. For control, the conA-tetramethylrhodamine solution was added to an empty well, and then removed and washed as described above. The conA fluorescence was detected at 555/580 nm using an Infinite M1000 (TECAN, Grödig, Austria) [54]. Experiments were made three times in three technological repeats. 

### 4.6. AI-2 Production Assay

Cultures of opportunistic pathogenic bacteria and conjugation mixture were used to prepare CFSs as stated above. The AI-2 production in the CFS was determined as described by [55]. Briefly, the reporter strain *V. harveyi* BB170 was grown overnight in AB medium at 30 °C with shaking at 120 rpm. On the next day, the reporter-strain culture was diluted 1:5000 in fresh AB medium and the CFSs of a 24 h conjugation mixture or single culture (control) was added at a concentration of 10% (*v/v*) and incubated for 5 h at 30 °C with shaking at 120 rpm. Positive and negative controls were a CFS of *V. harveyi* BB152 and AB medium, respectively. After the 5 h incubation, the bioluminescence of each sample was measured in a microplate reader Synergy H^1^ (BioTek, Santa Clara, CA, USA). AI-2 production in CFS was expressed as the percentage of specific bioluminescence induction of the *V. harveyi* BB170 reporter strain (relative light unit divided by OD_600_) compared to the positive control (100%). Experiments were made two times in three technological repeats.

### 4.7. Statistics

The results were statistically analyzed using Microsoft Office XP Excel and Statistica 6.0. The data are presented as *M ± SD* or median and quartiles. Student’s *t*-test (*t*-test) was used for statistical analysis of biofilm, exopolysaccharide matrix, CFU and AI-2 assay data and the Mann–Whitney test (U-test) was used for the statistical analysis of conjugative frequency data. The threshold for statistical significance was set at *p* < 0.05. Correlation analysis was performed using Pearson’s coefficient.

## 5. Conclusions

It is typical for *E. coli* to function as an integral part of a polymicrobial community attached to the surface in the host biotope. The efficiency of intraspecific conjugation is influenced by biotic factors, including the accompanying microbiota. Based on our results, we can conclude that interspecies interactions in dual-species biofilms formed by two strains of *E. coli* (conjugative plasmid donor and recipient) and representatives of three different taxa (*K. pneumoniae, E. faecalis* and *P. aeruginosa*) affect the conjugative transfer of the pOX38 plasmid into UPEC cells, reducing it by one to two orders of magnitude, regardless of mutual positive (*E. coli* and *K. pneumoniae*) or negative (*E. coli* and *P. aeruginosa*) influence during biofilm formation of co-culturing bacteria. Of note, complete inhibition of the plasmid transfer between *E. coli* was observed after exposure to *P. aeruginosa* CFS. The data obtained indicate a diverse response of bacterial cells in biofilms in symbiotic or antagonistic relationships, a response possibly regulated by AI-2. 

## Figures and Tables

**Table 1:** Frequency of the conjugative transfer of the pOX38 plasmid from the donor *E. coli* N4i pOX38 (D) to the recipient *E. coli* DL82 (R) within studied single- and dual-species biofilms and under influence of CFSs of opportunistic pathogenic bacterial species.

Conjugation Mixture	№	Frequency of Conjugation	
		R + D + Opportunistic Pathogenic Bacteria	R + D + CFSs of Opportunistic Pathogenic Bacteria
*E. coli* DL82 *+ E. coli* N4i pOX38	1	4.72 × 10^−4^ ± 2.28 × 10^−4^	
*E. coli* DL82 *+ E. coli* N4i pOX38*+ K. pneumoniae*	2	2.69 × 10^−4^ ± 1.01 × 10^−4^	4.93 × 10^−5^ ± 3.66 × 10^−5^ P_1–2_ = 0.0495 ^1^
*E. coli* DL82 *+ E. coli* N4i pOX38 *+ E. faecalis*	3	1.78 × 10^−5^ ± 5.38 × 10^−6^ P_1–3_ = 0.0495 P_2–3_ = 0.0495	1.93 × 10^−5^ ± 4.17 × 10^−7^ P_1–3_ = 0.0495
*E. coli* DL82 *+ E. coli* N4i pOX38*+ P. aeruginosa*	4	2.93 × 10^−5^ ± 3.07 × 10^−5^ P_1–4_ = 0.0495 P_2–4_ = 0.0495	0.00 × 10^0^ P_1–4_ = 0.0495 P_2–4_ = 0.0495 P_3–4_ = 0.0495

^1^ Statistically significant differences (P) between conjugation mixtures (designated №) as determined by *U*-test.

**Table 2:** The induction of luminescence of *Vibrio harveyi* reporter strain BB170 by CFSs of *V. harveyi* BB152, *E. coli*, opportunistic pathogenic bacteria and conjugation mixtures.

Cell-Free Supernatant (CSF) of Strains/Conjugation Mixtures	№	Induction of Luminescence, %
*V. harveyi* BB152	1	100.0
*E. coli* K12	2	386.8 ± 42.9 P_1–2_ = 0.012 ^1^
*E. coli* DL82	3	3.2 ± 0.7
*E. coli* N4i pOX38	4	1.3 ± 0.2
*E. coli* DL82 + *E. coli* N4i pOX38	5	73.7 ± 35.5 P_3–5_ = 0.027 P_4–5_ = 0.016
*K. pneumoniae*	6	190.1 ± 57.9 P_3–5_ = 0.023
*E. coli* DL82 + *E. coli* N4i pOX38+ *K. pneumoniae*	7	77.4 ± 42.3
*E. coli* DL82 + *E. coli* N4i pOX38 + CFS of *K. pneumoniae*	8	96.2 ± 6.3
*E. faecalis*	9	0.1 ± 0.05
*E. coli* DL82+ *E. coli* N4i pOX38 + *E. faecalis*	10	0.7 ± 0.1 P_5–10_ = 0.027
*E. coli* DL82+ *E. coli* N4i pOX38 + CFS of *E. faecalis*	11	37.0 ± 4.7 P_9–11_ = 0.005
*P. aeruginosa*	12	1.5 ± 1.0 P_2–12_ = 0.010
*E. coli* DL82 + *E. coli* N4i pOX38+ *P. aeruginosa*	13	23.5 ± 14.6 P_5–13_ = 0.040
*E. coli* DL82 + *E. coli* N4i pOX38+ CFS of *P. aeruginosa*	14	29.4 ± 8.9

^1^ Statistically significant differences (P) between assay variants (designated as №) as determined by *t*-test.

## Data Availability

All raw data are available on request from Marina V. Kuznetsova (mar@iegm.ru).

## References

[1] Besemer K. (2015). Biodiversity, community structure and function of biofilms in stream ecosystems. Res. Microbiol..

[2] Römling U., Kjelleberg S., Normark S., Nyman L., Uhlin B.E., Åkerlund B. (2014). Microbial biofilm formation: A need to act. J. Intern. Med..

[3] Mizan M.F., Jahid I.K., Ha S.D. (2015). Microbial biofilms in seafood: A food-hygiene challenge. Food Microbiol..

[4] Xu Y., Larsen L.H., Lorenzen J., Hall-Stoodley L., Kikhney J., Moter A., Thomsen T.R. (2017). Microbiological diagnosis of device-related biofilm infections. APMIS.

[5] Wagner E.M., Pracser N., Thalguter S., Fischel K., Rammer N., Pospíšilová L., Alispahic M., Wagner M., Rychli K. (2020). Identification of biofilm hotspots in a meat processing environment: Detection of spoilage bacteria in multi-species biofilms. Int. J. Food Microbiol..

[6] McLean R.J., Kakirde K.S. (2013). Enhancing metagenomics investigations of microbial interactions with biofilm technology. Int. J. Mol. Sci..

[7] Schulze A., Mitterer F., Pombo J.P., Schild S. (2021). Biofilms by bacterial human pathogens: Clinical relevance—Development, composition and regulation—Therapeutical strategies. Microb. Cell..

[8] Flores-Mireles A.L., Walker J.N., Caparon M., Hultgren S.J. (2015). Urinary tract infections: Epidemiology, mechanisms of infection and treatment options. Nat. Rev. Microbiol..

[9] Mancuso G., Midiri A., Gerace E., Marra M., Zummo S., Biondo C. (2023). Urinary tract infections: The current scenario and future prospects. Pathogens.

[10] Lila A.S.A., Rajab A.A.H., Abdallah M.H., Rizvi S.M.D., Moin A., Khafagy E.S., Tabrez S., Hegazy W.A.H. (2023). Biofilm lifestyle in recurrent urinary tract infections. Life.

[11] Oliveira A., Sousa J.C., Silva A.C., Melo L.D.R., Sillankorva S. (2018). Chestnut honey and bacteriophage application to control *Pseudomonas aeruginosa* and *Escherichia coli* biofilms: Evaluation in an ex vivo wound model. Front. Microbiol..

[12] Juarez G.E., Galván E.M. (2018). Role of nutrient limitation in the competition between uropathogenic strains of *Klebsiella pneumoniae* and *Escherichia coli* in mixed biofilms. Biofouling.

[13] Galván E.M., Mateyca C., Ielpi L. (2016). Role of interspecies interactions in dual-species biofilms developed in vitro by uropathogens isolated from polymicrobial urinary catheter-associated bacteriuria. Biofouling.

[14] Rendueles O., Travier L., Latour-Lambert P., Fontaine T., Magnus J., Denamur E., Ghigo J.M. (2011). Screening of *Escherichia coli* species biodiversity reveals new biofilm-associated antiadhesion polysaccharides. MBio.

[15] Zhu H., Liu H.J., Ning S.J., Gao Y.L. (2012). The response of type 2 quorum sensing in *Klebsiella pneumoniae* to a fluctuating culture environment. DNA Cell Biol..

[16] Brito P.H., Rocha E.P., Xavier K.B., Gordo I. (2013). Natural genome diversity of AI-2 quorum sensing in *Escherichia coli*: Conserved signal production but labile signal reception. Genome Biol. Evol..

[17] Li H., Li X., Wang Z., Fu Y., Ai Q., Dong Y., Yu J. (2015). Autoinducer-2 regulates *Pseudomonas aeruginosa* PAO1 biofilm formation and virulence production in a dose-dependent manner. BMC Microbiol..

[18] Yang Y., Li W., Hou B., Zhang C. (2018). Quorum sensing LuxS/autoinducer-2 inhibits *Enterococcus faecalis* biofilm formation ability. J. Appl. Oral. Sci..

[19] Wang Y.M., Dong W.L., Odah K.A., Kong L.C., Ma H.X. (2019). Transcriptome analysis reveals AI-2 relevant genes of multi-drug resistant *Klebsiella pneumoniae* in response to Eugenol at sub-MIC. Front. Microbiol..

[20] Chen L., Wilksch J.J., Liu H., Zhang X., Torres V.V.L., Bi W., Mandela E., Cao J., Li J., Lithgow T. (2020). Investigation of LuxS-mediated quorum sensing in *Klebsiella pneumoniae*. J. Med. Microbiol..

[21] Pereira C.S., Thompson J.A., Xavier K.B. (2013). AI-2-mediated signaling in bacteria. FEMS Microbiol. Rev..

[22] Laganenka L., Sourjik V. (2018). Autoinducer 2-dependent *Escherichia coli* biofilm formation is enhanced in a dual-species coculture. Appl. Environ. Microbiol..

[23] Yang L., Liu Y., Wu H., Hóiby N., Molin S., Song Z.J. (2011). Current understanding of multi-species biofilms. Int. J. Oral. Sci..

[24] Elias S., Banin E. (2012). Multi-species biofilms: Living with friendly neighbors. FEMS Microbiol Rev..

[25] Rendueles O., Ghigo J.M. (2012). Multi-species biofilms: How to avoid unfriendly neighbors. FEMS Microbiol. Rev..

[26] Antonova E.S., Hammer B.K. (2011). Quorum-sensing autoinducer molecules produced by members of a multispecies biofilm promote horizontal gene transfer to *Vibrio cholerae*. FEMS Microbiol. Lett..

[27] Hola V., Ruzicka F., Tenke P. (2011). The Formation of Poly-Microbial Biofilms on Urinary Catheters. Urinary Tract Infections.

[28] Reisner A., Höller B.M., Molin S., Zechner E.L. (2006). Synergistic effects in mixed *Escherichia coli* biofilms: Conjugative plasmid transfer drives biofilm expansion. J. Bacteriol..

[29] Koraimann G. (2018). Spread and persistence of virulence and antibiotic resistance genes: A ride on the F plasmid conjugation module. EcoSal Plus.

[30] Stephens C., Arismendi T., Wright M., Hartman A., Gonzalez A., Gill M., Pandori M., Hess D. (2020). F Plasmids are the major carriers of antibiotic resistance genes in human-associated commensal *Escherichia coli*. mSphere.

[31] Kuznetsova M.V., Maslennikova I.L., Pospelova J.S., Žgur Bertok D., Starčič Erjavec M. (2022). Differences in recipient ability of uropathogenic *Escherichia coli* strains in relation with their pathogenic potential. Infect. Genet. Evol..

[32] Smith R.S., Iglewski B.H. (2003). *P. aeruginosa* quorum-sensing systems and virulence. Curr. Opin. Microbiol..

[33] Laganenka L., Lee J.W., Malfertheiner L., Dieterich C.L., Fuchs L., Piel J., von Mering C., Sourjik V., Hardt W.D. (2023). Chemotaxis and autoinducer-2 signalling mediate colonization and contribute to co-existence of *Escherichia coli* strains in the murine gut. Nat. Microbiol..

[34] Tchebotar I.V., Mayanskiy A.N., Mayanskiy N.A. (2016). Matrix of microbial biofilms. Clin. Microbiol. Antimicrobial. Chemother..

[35] Sørensen S.J., Bailey M., Hansen L.H., Kroer N., Wuertz S. (2005). Studying plasmid horizontal transfer in situ: A critical review. Nat. Rev. Microbiol..

[36] Król J.E., Wojtowicz A.J., Rogers L.M., Heuer H., Smalla K., Krone S.M., Top E.M. (2013). Invasion of *E. coli* biofilms by antibiotic resistance plasmids. Plasmid.

[37] Cook L.C., Dunny G.M. (2014). The influence of biofilms in the biology of plasmids. Microbiol Spectr..

[38] Ferrières L., Hancock V., Klemm P. (2007). Specific selection for virulent urinary tract infectious *Escherichia coli* strains during catheter-associated biofilm formation. FEMS Immunol. Med. Microbiol..

[39] Keogh D., Tay W.H., Ho Y.Y., Dale J.L., Chen S., Umashankar S., Williams R.B.H., Chen S.L., Dunny G.M., Kline K.A. (2016). Enterococcal Metabolite Cues Facilitate Interspecies Niche Modulation and Polymicrobial Infection. Cell Host Microbe.

[40] Lopes S.P., Machado I., Pereira M.O. (2011). Role of planktonic and sessile extracellular metabolic byproducts on *Pseudomonas aeruginosa* and *Escherichia coli* intra and interspecies relationships. J. Ind. Microbiol. Biotechnol..

[41] Machado I., Lopes S.P., Sousa A.M., Pereira M.O. (2012). Adaptive response of single and binary *Pseudomonas aeruginosa* and *Escherichia coli* biofilms to benzalkonium chloride. J. Basic Microbiol..

[42] Cerqueira L., Oliveira J.A., Nicolau A., Azevedo N.F., Vieira M.J. (2013). Biofilm formation with mixed cultures of *Pseudomonas aeruginosa/Escherichia coli* on silicone using artificial urine to mimic urinary catheters. Biofouling.

[43] Castonguay M.H., van der Schaaf S., Koester W., Krooneman J., van der Meer W., Harmsen H., Landini P. (2006). Biofilm formation by *Escherichia coli* is stimulated by synergistic interactions and co-adhesion mechanisms with adherence-proficient bacteria. Res. Microbiol..

[44] Kuznetsova M.V., Maslennikova I.L., Karpunina T.I., Nesterova L.Y., Demakov V.A. (2013). Interactions of *Pseudomonas aeruginosa* in predominant biofilm or planktonic forms of existence in mixed culture with *Escherichia coli* in vitro. Can. J. Microbiol..

[45] Gritsenko V.A., Mrugova T.M., Kurlayev P.P., Belozertseva Y.P., Borisov S.D. (2016). Antagonistic relationship *Pseudomonas aeruginosa* with gram-negative bacteria. Bull. OSC.

[46] González Barrios A.F., Zuo R., Hashimoto Y., Yang L., Bentley W.E., Wood T.K. (2006). Autoinducer 2 controls biofilm formation in *Escherichia coli* through a novel motility quorum-sensing regulator (MqsR, B3022). J. Bacteriol..

[47] Liu C., Goh S.G., You L., Yuan Q., Mohapatra S., Gin K.Y., Chen B. (2023). Low concentration quaternary ammonium compounds promoted antibiotic resistance gene transfer via plasmid conjugation. Sci. Total Environ..

[48] Cho J., Jenneson S., Lane M., Macfadyen A., Van Rietschoten S. (2003). The effects of altering autoinducer-2 concentration on transfer efficiencies of the F and RPI plasmids to the Quorum sensing recipient *Escherichia coli* strain AB 1157. JEMI.

[49] Rijavec M., Rijavec M., Starčič Erjavec M., Ambrožič Avguštin J., Reissbrodt R., Fruth A., Križan-Hergouth V., Žgur-Bertok D. (2006). High prevalence of multidrug resistance and random distribution of mobile genetic elements among uropathogenic *Escherichia coli* (UPEC) of the four major phylogenetic groups. Curr. Microbiol..

[50] Starčič Erjavec M., Petkovšek Ž., Kuznetsova M.V., Maslennikova I.L., Žgur-Bertok D. (2015). Strain ŽP—The first bacterial conjugation-based “kill”-“anti-kill” antimicrobial system. Plasmid.

[51] Bassler B.L., Greenberg E.P., Stevens A.M. (1997). Cross-species induction of luminescence in the quorum-sensing bacterium *Vibrio harveyi*. J. Bacteriol..

[52] Guglielmetti E., Korhonen J.M., Heikkinen J., Morelli L., Von Wright A. (2009). Transfer of plasmid-mediated resistance to tetracycline in pathogenic bacteria from fish and aquaculture environments. FEMS Microbiol. Lett..

[53] Merritt J.H., Kadouri D.E., O’Toole G.A. (2005). Growing and analyzing static biofilms. Curr. Protoc. Microbiol..

[54] Surette M.G., Bassler B.L. (1998). Quorum sensing in *Escherichia coli* and *Salmonella typhimurium*. Proc. Natl. Acad. Sci. USA.

[55] Zorina A.S., Maksimova Y.G., Demakov V.A. (2019). Biofilm formation by monocultures and mixed cultures of *Alcaligenes faecalis* 2 and *Rhodococcus ruber* GT 1. Microbiology.

